# The Efficacy of Pharmacotherapy in the Treatment of Obesity in Patients With Type 2 Diabetes: A Systematic Review

**DOI:** 10.7759/cureus.65242

**Published:** 2024-07-24

**Authors:** Omar Alshahrani, Mohammed S Almalki

**Affiliations:** 1 Family Medicine, Prince Sultan Military Medical City, Riyadh, SAU

**Keywords:** weight loss, drug therapy, overweight, obesity, type 2 diabetes mellitus

## Abstract

Obesity is a global public health challenge that poses a significant threat to the effective control and management of type 2 diabetes mellitus (T2DM). Being overweight/obese with T2DM is associated with a wide range of comorbidities, including cardiovascular, cerebrovascular, and renal diseases. This systematic review aimed to investigate the drug therapy used globally among this type of patients in the period between 2014 and 2024. Four databases (PubMed, Web of Science, Scopus, and Cochrane) were searched using the keywords “(Drug Therapy OR Pharmaceutical Preparations OR Pharmacotherapy) AND (Diabetes Mellitus, Type 2) AND (Obesity OR Overweight OR Weight Loss OR Weight reduction) in the title and abstract. All papers assessing the efficacy of any drug class on blood sugar and body weight (BW) were included in the systematic review. Out of 5,206 papers extracted through the database search, 25 randomized clinical trials (RCTs) were considered suitable for the systematic review. The articles included 8,208 participants who tested different drug classes, e.g., glucagon-like peptide-1 (GLP-1) and sodium-glucose co-transporter-2 (SGLT2), with or without metformin. All the reviewed drugs showed significant weight loss over 12-52 weeks. However, the magnitude of weight loss was modest, and the long-term health benefits and safety remain unclear. Interventions that combine pharmacologic therapy with lifestyle modifications may be more effective but need additional research. Continued development of new treatment options for obesity in T2DM is crucial to reduce morbidity and mortality among these patients.

## Introduction and background

Strong evidence shows that greater weight loss often leads to more significant improvements in glycemic control and cardiovascular risk markers [1]. Numerous studies have stated that a 5% weight loss from baseline improves A1C by 0.5%, and a 15% weight loss from baseline improves A1C by ~1% [2-4].

Unfortunately, achieving both weight and glycemic control is challenging. Lifestyle modifications alone might lead to initial weight loss, but weight regain is common [5]. In addition, some anti-hyperglycemic agents (AHAs), e.g., thiazolidinedione, sulfonylurea, and glinide, are also associated with weight gain [6-8]. Therefore, there has been an increased need for newer modalities that address these unmet clinical needs.

Metformin has been used as the first line of treatment for type 2 diabetes mellitus (T2DM) since it was approved by the Food and Drug Administration (FDA) in 1994 [9]. Despite its efficacy and safety, recent clinical trials have been investigating the potency of newer AHAs such as dipeptidyl peptidase-4 (DPP-4) inhibitors, sodium-glucose co-transporter-2 (SGLT2) inhibitors, and glucagon-like peptide-1 (GLP-1) receptor agonists on weight loss and glycemic control alone or in combination with behavioral therapy and metformin when treatment escalation is indicated [10,11]. The effects of other drug classes such as pegbelfermin (a polyglycated analog of human fibroblast growth factor 21 (FGF21)), montelukast (leukotriene receptor antagonist), phentermine and topiramate (anorectics and anticonvulsants), bimagrumab (human monoclonal antibody inhibitor of activin type II receptors (ActRII)), and lorcaserin (serotonin receptor antagonist) have also been studied.

Because of the rising necessity of finding suitable treatment options to prevent the complications and comorbidities of overweight/obese patients with T2DM, we conducted a comprehensive overview of all the pharmacotherapy used in the management of patients with coexisting obesity and T2DM in the last 10 years. This systematic review of the available literature will provide better guidance for physicians in making personalized treatment plans and improving the lives of these patients.

## Review

This systematic review complied with established criteria (Preferred Reporting Items for Systematic Reviews and Meta-Analyses (PRISMA)) [12,13].

Search strategy

The systematic review was conducted through a thorough literature search of PubMed, Scopus, Web of Science, and Cochrane library databases using the keywords in the abstract and title: (Drug Therapy OR Pharmaceutical Preparations OR Pharmacotherapy) AND ((Diabetes Mellitus, Type 2) AND (Obesity OR Overweight OR Weight Loss OR Weight reduction OR weight change OR body weight)). One researcher screened studies published from 2014 to 2024 examining the drug therapy used with obesity in T2DM to select studies that matched the inclusion and exclusion criteria. Then, key data points were retrieved from the final record of the included research.

Inclusion and exclusion criteria

All randomized clinical trials (RCTs) assessing the efficacy of different drug classes in the treatment of obesity associated with T2DM were included in the systematic review. We excluded study designs other than RCTs, animal studies, non-English studies, studies assessing obesity in other types of diabetes, individuals < 18 years, body mass index (BMI) < 25 kg/m^2^, patients improving on dietary modifications only, duplicated papers, studies published before 2014 or conducted on the timeframe for obesity in T2DM before 2014, unpublished studies, studies with insufficient data or findings, studies with irrelevant findings, studies that did not include clinical samples, and studies for which full text was unavailable.

Screening and data extraction

Endnote software (Clarivate Analytics, PA, USA) [14] removed duplicates. The retrieved references were screened in two steps: the first consisted of screening the titles/abstracts independently by two authors to determine their relevance, and the second consisted of screening the full-text papers and evaluating them for inclusion criteria. Independent authors independently extracted data in a Microsoft Excel spreadsheet (Microsoft Corp., Redmond, WA). The data included authors, year of publication, study design and period, objective, methodology, population characteristics, and results of different drug classes used for the treatment of obesity with T2DM. Any discrepancies identified were thoroughly elaborated among the authors until a consensus was reached. The Rayyan website was used in the selection process [15].

Risk of bias assessment

For data synthesis, a summary table was created using data from relevant studies to provide a qualitative interpretation of the findings and study components. The Cochrane risk of bias assessment tool 2 (RoB2) was utilized to evaluate the quality of RCTs [16]. The RoB2 tool assesses the risk of bias (ROB) based on the following domains: randomization process, deviation from intended interventions, outcome measurement, missing outcome data, selection of reported results, and the potential sources of bias. The outcome assessed was weight loss in overweight/obese patients with T2DM during 2014-2024. The judgment options were low, moderate, and high, and the overall ROB was reached using signaling questions. Two independent authors conducted the ROB, and disagreements were resolved by discussing them with a third author.

Results

**Figure 1:**
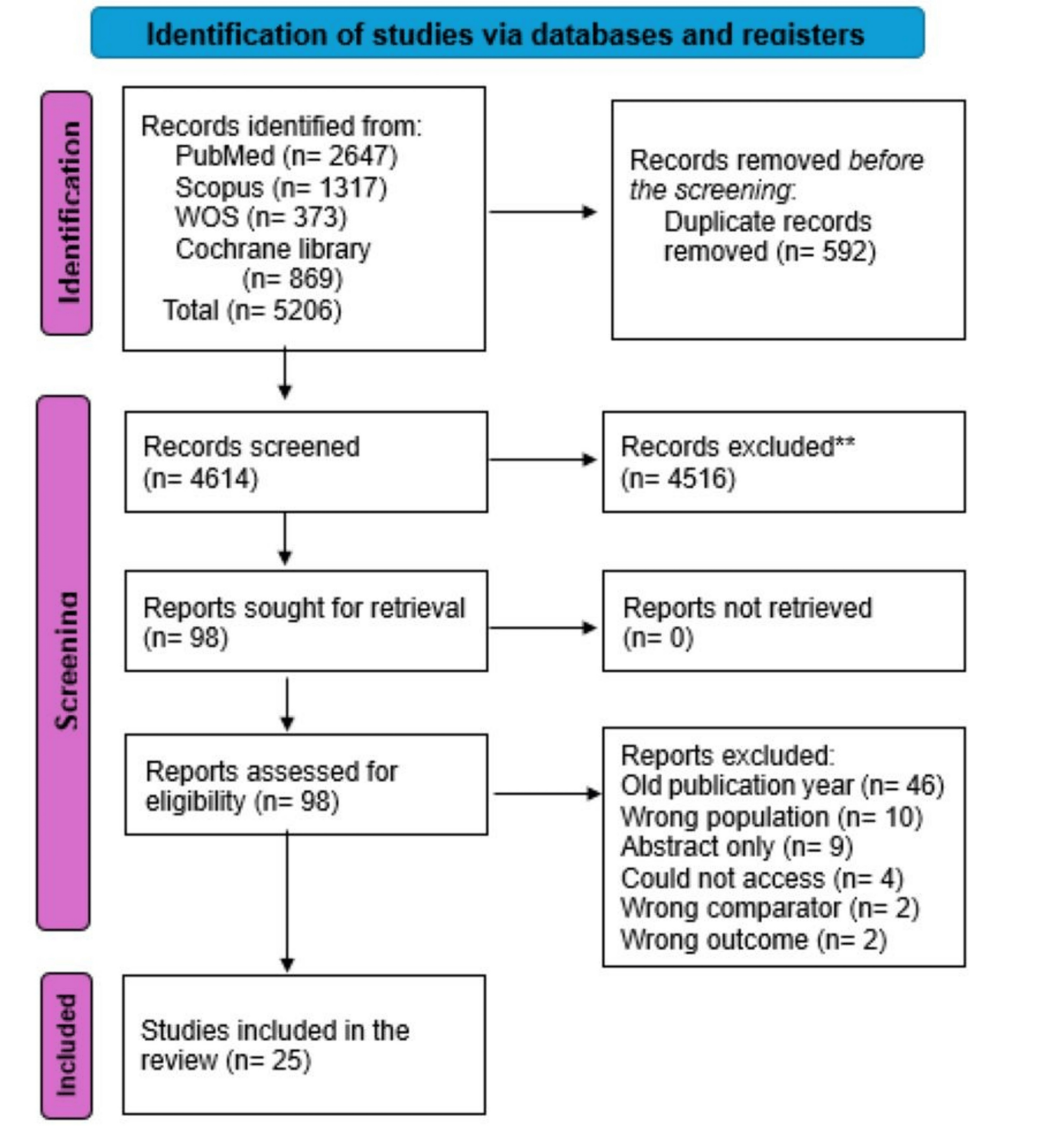
Flow diagram of study selection for the systematic review WOS: Web of Science

A total of 5,206 papers were extracted from four databases (PubMed, Web of Science, Scopus, and Cochrane). Of these, 592 were omitted as duplicates via EndNote (Clarivate Analytics, Philadelphia, PA). After a thorough full-text screening, we identified 26 RCTs that met our inclusion criteria for this systematic review, as shown in Figure 1.

Overview of the included studies

The included papers were published between 2014 and 2024 in different countries across the world. The studies' duration range was six to 56 weeks (Table 1). The study design was limited to RCTs. The articles included 8,208 individuals, mostly males, with an age average of 54.5±8.7, BMI ranging from 27 to 40 kg/m^2^, diagnosed with T2DM inadequately controlled with diet and exercise (hemoglobin A1C (HbA1C): 6.5%-8.5%). Most patients were already taking at least one hypoglycemic agent, e.g., metformin and sulfonylurea, at the beginning of the study but were not controlled. Most RCTs excluded patients with type 1 diabetes, a history or presence of a condition that could interfere with the assessment of the study drug (as judged by investigators), fasting blood glucose (FBG) concentration of 11.1 mmol/L or more, concurrent or previous use of a GLP-1 agonist within three months prior to screening, ongoing hypocaloric diet, or use of weight-loss agents or insulin for glycemic control within 12 months prior to screening and women who were lactating or of childbearing potential. All patients included in the RCTs were advised to follow a restricted diet and exercise program along with the tested drugs.

**Table 1:** Characteristics of the included studies SD: standard deviation, BMI: body mass index, HbA1C: hemoglobin A1C, GLP-1: glucagon-like peptide-1, GLP-1R: glucagon-like peptide-1 receptor, GCGR: glucagon receptor, BW: body weight, TEAE: treatment-emergent adverse event, biw: twice a week, qw: once a week, GLP-1RA: glucagon-like peptide-1 receptor agonists, WC: waist circumference, VFA: visceral fat area, FPG: fasting plasma glucose, SGLT2: sodium-glucose co-transporter-2, FGF21: fibroblast growth factor 21, OGTT: oral glucose tolerance test; WHR: WC-to-height ratio, FSG: fasting serum glucose, OADs: oral antidiabetic drugs, SBP: systolic blood pressure, LM: lean mass, TG: triglyceride, HDL-C: high-density lipoprotein cholesterol, BID: two times a day, HOMA-IR: Homeostatic Model Assessment for Insulin Resistance, 2hPBG: two-hour postprandial blood glucose, FINS: fasting insulin, VAT: visceral adipose tissue, FMI: fat mass index, SMI: skeletal mass index, FFMI: fat-free mass index, QD: once a day, UGE2: urinary glucose excretion, ActRII: activin type II receptors

Author, publication year, location	Population size and characteristics	Drug name, class, and dose	Study duration	Relevant outcome measures	Adverse effects
Ambery et al. (2018), Germany [17]	Intervention: n=25, control: n=26; age (mean±SD): 56±7.2 years; male (number (%)): 13 (52%); BMI (mean±SD): 32±4.4 kg/m^2^; HbA1C (mean±SD): 7.2±0.6%	Cotadutide (GLP-1/GCGR agonist), 200 μg	6 weeks	Change in BW and HbA1C from baseline to the end of treatment	Any TEAE: n=22 (88%); any serious TEAE: n=0; gastrointestinal disorders total: n=18 (72%); nausea: n=13 (52%); vomiting: n=8 (32%)
Asano et al. (2023), Japan [18]	Intervention: n=12, control: n=4; age (median (range)): 58.5 (34-69) years; male (number (%)): 7 (58.3%); BMI (median (range)): 27.185 (25.21-34.71) kg/m^2^; HbA1C (mean±SD): 7.41±0.67%	Cotadutide (GLP-1/GCGR agonist), 600 μg.	10 weeks	Percentage change in BW and the proportion of patients achieving 5% or greater BW loss by the end of the extension period; change in HbA1C from baseline to the end of treatment	Any TEAE: n=11 (91.7%); any serious TEAE: n=0; gastrointestinal disorders total: n=9 (75%); nausea: n=8 (66.7%); vomiting: n=1 (8.3%)
Blüher et al. (2024), Germany [19]	Intervention: survodutide: n=49, semaglutide: n=50, control: n=59; age (mean±SD): survodutide: 5.7±9.4 years, semaglutide: 55.8±10.5 years; male (number (%)): survodutide: 27 (55.1%), semaglutide: 34 (68%); BMI (mean±SD): survodutide: 34.9±7 kg/m^2^, semaglutide: 33.4±6.1 kg/m^2^; HbA1C (mean±SD): survodutide: 7.97±0.71%, semaglutide: 8.03±0.82%	Survodutide (GCGR/GLP-1R): 1.8 mg biw; semaglutide (GLP-1): 1 mg qw	16 weeks	Absolute change in HbA1C and relative change in BW from baseline to the end of treatment	Any TEAE: n=42 (85.7%); semaglutide: 26 (52%); any serious TEAE: n=0; gastrointestinal disorders total: n=50%; semaglutide: NA; nausea: n=22 (44.9%), semaglutide: 6 (12%); vomiting: n=10 (20.4%); Semaglutide: 2 (4)
Cai et al. (2023), China [20]	Intervention: n=105, control: n=52; age (mean±SD): 4.3±10 years; male (number (%)): 67 (64.4); BMI (mean±SD): 30.0±3.6 kg/m^2^; HbA1C (mean±SD): 8.79±0.83%	Polyethylene glycol loxenatide (GLP-1RA), 0.3mg	16 weeks	Change in BW, the proportion of patients with ≥5% and ≥10% weight loss, BMI, WC, and VFA; changes in HbA1C and FPG from baseline to the end of treatment	Any TEAE: n=48 (46.2%); any serious TEAE: n=3 (2.9%); gastrointestinal disorders total: n=25 (24%); nausea: n=13 (12.5%); vomiting: n=6 (5.8%)
Cefalu et al. (2015) [10]	Intervention: canagliflozin 100 mg: n=812, canagliflozin 300 mg: n=812, control: n=626; age (mean±SD): 55.9±9.8 years; male (number (%)): 396 (48.8%); BMI (mean±SD): 32.2±6.45 kg/m^2^; HbA1C (mean±SD): 8±0.95%	Canagliflozin (SGLT2 inhibitor) 100 and 300 mg	26 weeks	Percentage change in BW and the proportion of patients achieving 5% or greater BW loss by the end of the extension period; change in HbA1C from baseline to the end of treatment	NA
Charles et al. (2019), USA and Canada [21]	Intervention: n=96, control: n=24; age (mean±SD): 56±10 years; male (number (%)): 53 (55%); BMI (mean±SD): 35±4 kg/m^2^; HbA1C (mean±SD): 7.8±1.0%	Pegbelfermin (PEGylated FGF21), 20 mg QD	12 weeks	Change in BW, WC, and BMI; change in HbA1C and insulin sensitivity (fasting and following OGTT)	Any TEAE: n=61 (64%); any serious TEAE: n=1 (4%); nausea: n=6 (6%); vomiting: n=2 (2%)
Davies et al. (2015), 9 countries (France, Germany, Israel, South Africa, Spain, Sweden, Turkey, United Kingdom (England and Scotland only), and USA) [22]	Intervention: liraglutide 3 mg (n=423), liraglutide 1.8 mg: n=212, control: n=211; age (mean±SD): 55.0±10.8 years; male (number (%)): 220 (52%); BMI (mean±SD): 37.1±6.5 kg/m^2^; HbA1C (mean±SD): 7.9±0.8%	Liraglutide (GLP-1RA) 1.8 mg and 3 mg	56 weeks	NA	Any TEAE: liraglutide 3 mg: n=392 (92.9%), liraglutide 1.8 mg: n=190 (90.5); any serious TEAE: liraglutide 3 mg: n=37 (8.8%), liraglutide 1.8 mg: n=18 (8.6%); gastrointestinal disorders total: liraglutide 3 mg: n=275 (65.2%), liraglutide 1.8 mg: n=118 (56.2%); nausea: liraglutide 3 mg: n=138 (32.7%), liraglutide 1.8 mg: n=66 (31.4%); vomiting: liraglutide 3 mg: n=66 (15.6%), liraglutide 1.8 mg: n=21 (10)
Di Prospero et al. (2021), USA [23]	Intervention: efinopegdutide 5 mg: n=48, 7.4 mg: n=49, 10 mg: n=49; control: n=49; age (mean±SD): 56.3±9 years; male (number (%)): 19 (39.7%); BMI (mean±SD): 40.6±4 kg/m^2^; HbA1C (mean±SD): 7.6±0.86%	Efinopegdutide 5 mg, 7.4 mg, or 10 mg (GLP-1/GCGR agonist)	10 weeks	The percent change in BW from baseline to the end of treatment; proportion of participants achieving ≥5% and ≥10% weight loss; change in HbA1C, FPG, and fasting plasma insulin	Any TEAE: 5 mg: n=30 (62.5%), 7.4 mg: n=39 (79.6%), 10 mg: n=36 (73.5%); any serious TEAE: 5 mg: n=2 (4.2%), 7.4 mg: n=1 (2%), 10 mg: n=3 (6.1%); gastrointestinal disorders total: 5 mg: n=19 (39.6%), 7.4 mg: n=24 (49%), 10 mg: n=27 (55.1%); nausea: 5 mg: n=13 (27.1%); 7.4 mg: n=17 (34.7%); 10 mg: n=21 (42.9%); vomiting: 5 mg: n=8 (16.7%), 7.5 mg: n=12 (24.5%), 10 mg: n=17 (34.7%)
El-Khateeb et al. (2023), Egypt [24]	Intervention: n=50, control: n=50; age (mean±SD): 45.48±4.21 years; male (number (%)): 25 (50%); BMI (mean±SD): 32.45±0.89 kg/m^2^; HbA1C (mean±SD): 8.56±0.47%	Montelukast (leukotriene receptor antagonist montelukast), 10 mg/day plus 2 g/day metformin	12 weeks	Changes in BW, BMI, and HbA1C from baseline to the end of treatment	Nausea: n=3 (6.98%); vomiting: n=3 (6.98%); abdominal distension: n=8 (18.6%)
Feng et al. (2015), China [25]	Intervention: n=328; age (mean±SD): 47±11 years; male (number (%)): 238 (72.56%); BMI (mean±SD): 29.6±3.8 kg/m^2^; HbA1C (mean±SD): 8.1±1.6%	Liraglutide (GLP-1RA), 1.8 mg	24 weeks	Changes in BW, BMI, WC, and WHR; changes in HbA1C, fasting and postprandial blood glucose, and islet function	Any TEAE: n=71 (94.7%); any serious TEAE: n=4 (5.3%); nausea: n=9 (12%)
Frias et al. (2019) [26]	Intervention: 1.5 mg: n=81, 3 mg: n=79, 4.5 mg: n=76, control: n=82; age (mean±SD): 57±10 years; male (number (%)): 110 (46%); BMI (mean±SD): 32.2±4.8 kg/m^2^; HbA1C (mean±SD): 8.0±0.8%	Dulaglutide (GLP-1), 1.5 mg, 3 mg, 4.5 mg	18 weeks	Reduction of HbA1C; change from baseline in BW and FSG	Any TEAE: 1.5 mg: n=54 (66.7%), 3 mg: n=66 (83.5%), 4.5 mg: n=53 (69.7%); any serious TEAE: 1.5 mg: n=3 (3.7%), 3 mg: n=5 (6.3%), 4.5 mg: n=2 (2.6%); gastrointestinal disorders total: 1.5 mg: n=35 (43.2%), 3 mg: n=39 (49.4%), 4.5 mg: n=36 (47.4%); nausea: 1.5 mg: n=18 (22.2%), 3 mg: n=19 (24.1%), 4.5 mg: n=23 (30.3%); vomiting: 1.5 mg: n=9 (11.1%), 3 mg: n=8 (10.1%), 4.5 mg: n=10 (13.2%)
Garvey et al. (2014) [27]	Intervention: n=75, control: n=55; age (mean±SD): 49.7±7.5years; male (number (%)): 17 (22.67%); BMI (mean±SD): 35.5±4.7 kg/m^2^; HbA1C (mean±SD): 8.8±1.2%	Phentermine/topiramate (anorectics and anticonvulsants), 15 mg/92 mg	56 weeks	Change in HbA1C levels; percentage of weight loss, percentage of subjects achieving HbA1C levels of ≤7% and ≤6.5%, changes in concomitant use of antidiabetic medications, and changes in fasting glucose, fasting insulin levels, and insulin sensitivity	Any TEAE: n=71 (94.7%); any serious TEAE: n=4 (5.3%); paresthesia: n=15 (20%); constipation: n=10 (13.3%)
Garvey et al. (2020) [28]	Intervention: n=198, control: n=198; age (mean±SD): 55.9±11.3 years; male (number (%)): 90 (45.5%); BMI (mean±SD): 35.9±6.5 kg/m^2^; HbA1C (mean±SD): 7.9±1.1%	Liraglutide (GLP-1), 3 mg	56 weeks	Efficacy of the drug on weight loss; efficacy in individuals treated with basal insulin and up to two OADs	Any TEAE: n=180 (92.3%); any serious TEAE: n=16 (8.2%); gastrointestinal disorders total: n=121 (62.1%); nausea: n=58 (29.7%); vomiting: n=32 (16.4%)
Heymsfield et al. (2020) [29]	Intervention: 5 mg: n=466, 15 mg: n=463, control: n=448; age (mean±SD): 57.0±9.5 years; male (number (%)): 241 (52.1%); BMI (mean±SD): 32.6±5.5 kg/m^2^; HbA1C (mean±SD): 8.1±0.9%	Ertugliflozin (SGLT) inhibitor, 5 mg and 15 mg	26 weeks	Percent change in HbA1C, FPG, BW, and SBP from baseline to the end of treatment at week 26	Any TEAE: 5 mg: n=208 (44.6%), 15 mg: n=232 (50.1%); any serious TEAE: 5 mg: n=13 (2.8%), 15 mg: n=9 (1.9%)
Heymsfield et al. (2021), USA and United Kingdom [30]	Intervention: n=37, control: n=38; age (mean±SD): 60.7±7.5 years; male (number (%)): 14 (38%); BMI (mean±SD): 32.7±3.2 kg/m^2^; HbA1C (mean±SD): 7.99±1.03%	Bimagrumab, a human monoclonal antibody inhibitor of ActRII (10 mg/kg up to 1,200 mg in 5% dextrose solution)	48 weeks	Least square mean change from baseline to week 48 in total body fat mass, BW, LM, and WC; changes in HbA1C level from baseline to week 48	Any TEAE: n=31 (84%); any serious TEAE: n=3 (8%); nausea: n=4 (11%); diarrhea: n=15 (41%)
Hollander et al. (2021) [31]	Intervention: n=149, control: n=143; age (mean±SD): 53±10 years; male (number (%)): 63 (42%) ; BMI (mean±SD): 36.9±5 kg/m^2^; HbA1C (mean±SD): 7.54±0.84%	Taspoglutide (GLP-1), 20 mg	24 weeks	Mean absolute change from baseline in HbA1C at the end of treatment (week 24); changes in BW, anthropometric measurements (waist and hip circumference and waist-to-hip ratio), and HbA1C response rates (percentage of patients with HbA1C 6.5% and 7%)	Any TEAE: n=122 (79.2%); any serious TEAE: n=10 (6.5%); nausea: n=54 (35.1%); vomiting: n=37 (24%)
Kato et al. (2017), Japan [32]	Intervention: n=56; age (mean±SD): 48.7±11.5 years; male (number (%)): 12 (44%); BMI (mean±SD): 30.3±5.3 kg/m^2^; HbA1C (mean±SD): 8.7±1.9%	Dapagliflozin (SGLT) inhibitors, 5 mg	24 weeks	Changes from baseline in HbA1C and body composition; changes in VFA and subcutaneous fat area after 12 weeks	NA
Li et al. (2022), China [33]	Intervention: n=20, control, n=20; age (mean±SD): 63.29±1.27 years; male (number (%)): 10 (50%); BMI (mean±SD): 28.19±3.23 kg/m^2^; FPG (mean±SD): 6.2±1.8 mmol/L	Polyethylene glycol loxenatide (long-acting GLP-1RA), weekly 100 μg injections	12 weeks	Changes in FPG; changes in BW and BMI; changes in lipid profile (TG and HDL-C)	NA
Nahra et al. (2021), Bulgaria, Canada, Czech Republic, Germany, Mexico, Russia, Slovakia, and USA [34]	Intervention: 100 μg: n=100, 200 μg: n=256, 300 μg: n=256, liraglutide 1.8 mg: n=110 control: n=112; age (mean±SD): 56.7±9.9 years; male (number (%)): 329 (45.5%); BMI (mean±SD): 35.3±5.4 kg/m^2^; HbA1C (mean±SD): 8.1±1.1%	Cotadutide, a dual GLP-1/GCGR agonist, 100 μg, 200 μg, and 300 μg	54 weeks	Changes in HbA1C and proportion of participants achieving the target HbA1C levels <7% (53 mmol/mol); absolute change in BW, percent change in BW, and proportion of participants achieving weight loss ≥5% and ≥10%	Any TEAE: cotadutide 100 μg: n=73 (73%), cotadutide 200 μg: n=202 (78.9%), cotadutide: 300 μg: n=206 (80.5%), liraglutide: 1.8 mg: n=68 (61.8%); any serious TEAE: cotadutide 100 μg: n=12 (12%), cotadutide 200 μg: n=33 (12.9%), cotadutide 300 μg: n=20 (7.8%), liraglutide 1.8 mg: n=8 (7.3%); gastrointestinal disorders total: cotadutide 100 μg: n=41 (41%), cotadutide 200 μg: n=149 (58.2%), cotadutide 300 μg: n=152 (59.4%), liraglutide 1.8 mg: n=30 (27.3%); nausea: cotadutide 100 μg: n=23 (23%), cotadutide 200 μg: n=85 (33.2%), cotadutide 300 μg: n=105 (41%), liraglutide 1.8 mg: n=17 (15.5%); vomiting: cotadutide: 100 μg: n=10 (10%), cotadutide 200 μg: n=51 (19.9%), cotadutide 300 μg: n=43 (16.8%), liraglutide 1.8 mg: n=3 (2.7%)
Neeland et al. (2016) (Cohort 1), USA [35]	Intervention: n=552, control: n=271; age (mean±SD): 60.2±9.1 years; male (number (%)): 327 (59.2); BMI (mean±SD): 32.7±5.2 kg/m^2^; HbA1C (mean±SD): 7.90±0.74%	Empagliflozin (SGLT) inhibitor, 10 mg or 25 mg	12 weeks	Changes in weight, WC, estimated total body fat, index of central obesity, and visceral adiposity index	NA
Okanović et al. (2014), Tešanj, Bosnia, and Herzegovina [36]	Intervention: n=30, control: n=30; age (mean±SD): 62.97±1.47 years; male (number (%)): 15 (50%), BMI (mean±SD): 30.57±0.46 kg/m^2^; FPG (mean±SD): 8.16±0.12 mmol/L	Alpha-lipoic acid (caprylic acid-derived antioxidant), 600 mg/day	20 weeks	Changes in BMI and serum concentration of glucose, cholesterol, and triglycerides	NA
Pi-Sunyer et al. (2016), USA [37]	Intervention: n=94, control: n=271; age (mean±SD): 55.0±7.38 years; male (number (%)): 45 (47.9%); BMI (mean±SD): 35.7±4.78 kg/m^2^; HbA1C (mean±SD): 7.9±0.80%	Lorcaserin (serotonin receptor agonists), 10 mg BID	52 weeks	Proportion of patients achieving ≥5% weight, absolute change in BW, and proportion of patients achieving ≥10% weight loss from baseline to week 52; changes from baseline in lipid levels, blood pressure, HbA1C, FPG, and HOMA-IR to week 52	NA
Song et al. (2023), China [38]	Intervention: n=50, control: n=50; age (mean±SD): 51.38±6.39 years; male (number (%)): 26 (52%); BMI (average (range)): 28.50 (25.25-30.10) kg/m^2^; HbA1C (average (range)): 7.60% (6.90%-8.13%)	Polyethylene glycol loxenatide (GLP-1RA)	24 weeks	Change in weight and BMI; change in FPG, 2hPBG, FINS, HbA1C, and HOMA-IR from baseline to the end of treatment	NA
Volpe et al. (2022) [39]	Intervention: n=180; age (mean±SD): 64.9±10.8 years; male (number (%)): 108 (60.2%); BMI (mean±SD): 38.8±7.7 kg/m^2^; HbA1C (mean±SD): 52.9±21.6 mmol/mol	Semaglutide (GLP-1RA), once weekly	24 weeks	Change in BW, BMI, and WC; change in the VAT, FMI, SMI, and FFMI	NA
He et al. (2019) [40]	Intervention: n=44, control: n=44; age range: (40-56) years; male (range): 43%-50%; BMI (range): 30.2-40.2 kg/m^2^; HbA1C (mean±SD): 8.2±0.7%	Licogliflozin (SGLT-1/2) inhibitor, 150 mg QD	12 weeks	Change in BW and blood glucose; effects on UGE2 and incretin hormones	Any TEAE: n=43 (97.7%); nausea: n=8 (18.2%); diarrhea: n=40 (90.9%)

Moreover, all patients were measured for BW, BMI, waist circumference (WC), HbA1C, FBG, and lipid profile at the beginning of RCTs and at one or more points during the study. The major drug class studied in the included RCTs was glucagon-like peptide-1 receptor agonists (GLP-1RAs), e.g., semaglutide, taspoglutide, cotadutide, survodutide, polyethylene glycol loxenatide (PEG-Loxe), liraglutide, and efinopegdutide, followed by SGLT-1 inhibitors, e.g., canagliflozin, ertugliflozin, dapagliflozin, and licogliflozin. Among the tested drugs were pegbelfermin, montelukast, phentermine (PHEN) and topiramate (TPM), bimagrumab, alpha-lipoic acid (a caprylic acid-derived antioxidant), and lorcaserin.

These drugs were assessed according to their potency in lowering blood glucose levels by measuring the change in the HbA1C and FBG, as well as their efficacy in inducing weight loss, evidenced by the reduction in BW, BMI, WC, and body fat percent. The adverse effects of each drug were also reported. The results showed the superiority of these drugs (whether used alone or in combination with metformin) in controlling blood glucose and BW over lifestyle modifications. The most reported side effects were gastrointestinal disorders, including nausea and vomiting.

Risk of bias assessment

**Figure 2:**
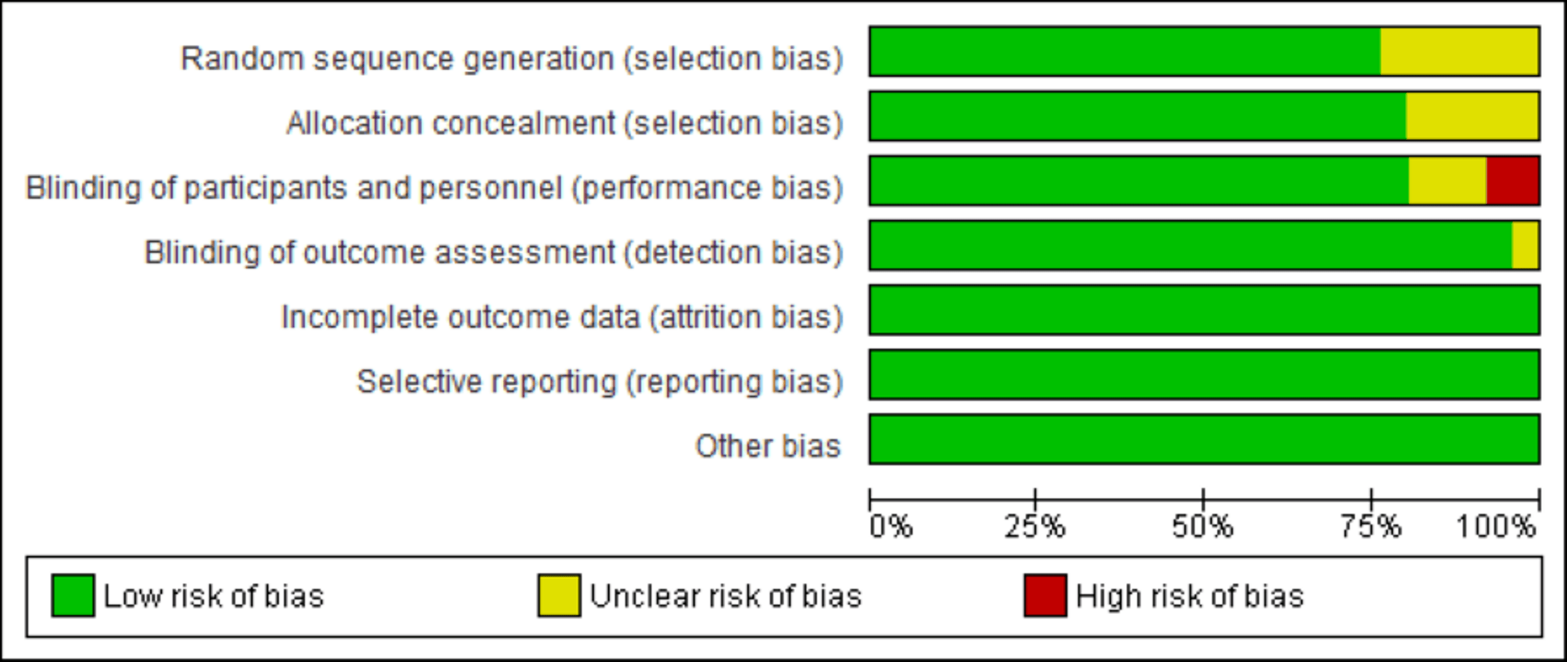
Risk of bias graph Review authors' judgments about each risk of bias item presented as percentages across all included studies

**Figure 3:**
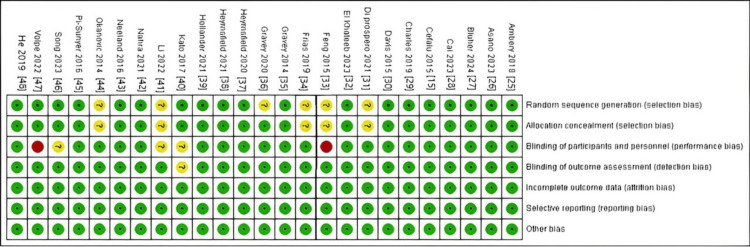
Risk of bias summary Review authors' judgments about each risk of bias item for each included study [10,17-40]

Using the Cochrane RoB2 tool, 25 RCTs were assessed as having a low ROB, including nine RCTs with unclear risk in the domain of randomization method, allocation, and blinding of participants. However, the overall ROB is low. All details regarding the ROB assessment are illustrated in Figure 2 and Figure 3.

In this systematic review, we summarized and synthesized the most up-to-date data from RCTs that evaluate the efficacy of recent drugs used to treat obesity associated with T2DM. To the best of our knowledge, there was no updated systematic review of the pharmacotherapy used in the last decade for the treatment of overweight/obese patients with T2DM. According to older studies, drugs such as fluoxetine, orlistat, and sibutramine were investigated. However, they only caused modest weight loss. In addition, their long-term health benefits and safety remained unclear [41]. Based on recent RCTs, the most promising two drug classes were GLP-1RAs and SGLT2 inhibitors [42]. Other drugs, classes, FGF21 analogs, leukotriene receptor antagonists, anorectics and anticonvulsants, serotonin receptor antagonists, and antioxidants were explored.

GLP-1RAs achieve glycemic control through mechanisms such as boosting insulin secretion induced by hyperglycemia, decreasing glucagon secretion during hyperglycemia, slowing gastric emptying, and preventing significant increases in postprandial glucose [43,44]. SGLT inhibitors work by blocking SGLT2 cotransporters in the proximal tubules in the kidney, leading to the inhibition of glucose re-absorption and promotion of the renal excretion of glucose, thereby modestly lowering elevated blood glucose levels [45]. Despite their efficacy in controlling blood glucose levels, their effect on reduction in BW and BMI was still uncertain. There are a wide variety of GLP-1RAs and SGLT2 inhibitors, including short-acting and long-acting drugs. Because of these differences in pharmacokinetics, efficacy, adverse reaction rates, and dosing requirements of each GLP-1RA and SGLT2 inhibitor, most RCTs focused on evaluating each drug independently.

GLP-1 drugs

Cotadutide

Three RCTs were carried out by Asano et al. (2023) [18], Nahra et al. (2021) [34], and Ambery et al. (2018) [17] to investigate the effects of cotadutide on BW and blood glucose versus placebo. The results showed that the drug significantly reduced the HbA1C and BW at the end of the treatment. In the study by Asano et al. (2023) [18], the mean respective changes in HbA1C were 1.13% versus 0.17% in placebo, and the mean percentage changes in BW were 6.93% versus 1.23% in placebo. Also, cotadutide was tolerated up to 600 μg. According to Nahra et al. (2021) [34], the most common adverse events with cotadutide (nausea: 35%, vomiting: 17%) decreased over time.

Survodutide

Blüher et al. (2024) [19] proved that survodutide dose-dependently reduced HbA1C after 16 weeks of treatment by up to -18.72 mmol/mol (-1.71%) versus semaglutide -16.07 mmol/mol (-1.47%) and placebo (-1.62 mmol/mol (-3.83, 0.59); (p<0.0001)). Furthermore, survodutide at doses ≥ 1.8 mg qw induced greater BW reductions than semaglutide (up to -8.7% (8.4 kg) dose group 6 (DG6) versus -5.3% (5.2 kg) semaglutide; p<0.001). The adverse effects were mainly gastrointestinal and were dose-related [19].

Semaglutide

A single-arm, open-label study by Volpe et al. (2022) [39] showed that the drug improved HbA1C (-11.16±2.99; p<0.01) and caused a significant decrease in BW (-9.89±0.99 kg; p<0.01) at all observational points.

Polyethylene Glycol Loxenatide

Three RCTs by Cai et al. (2023) [20], Song et al. (2023) [38], and Li et al. (2021) [33] revealed that PEG-Loxe along with lifestyle interventions or oral antidiabetic drug therapy has significantly decreased BW, HbA1C, fasting plasma glucose (FPG), low-density lipoprotein cholesterol (LDL-C), total cholesterol (TC), and triglyceride (TG) levels in the combined treatment group more than that in the control group in addition to an increase in the level of high-density lipoprotein cholesterol (HDL-C) (p<0.05) compared to metformin alone.

According to Cai et al. (2023) [20], weight loss was 7.52 kg (8.37%) with PEG-Loxe versus 2.96 kg (3%) with metformin (p<0.001). In addition, a higher proportion of patients lost ≥5% (61.5% versus 25%) or 10% (26.9% versus 5.8%) BW in the PEG-Loxe group than in the metformin group (p<0.01).

Liraglutide

Three RCTs, Davies et al. (2015) [22], Feng et al. (2015) [25], and Garvey et al. (2020) [28] showed that in addition to blood glucose control, there was a significant weight loss between the liraglutide group and the control group.

According to Garvey et al. (2020) [28], the estimated treatment difference in BW loss was 24.3% (95% CI: 25.5, 23.2); p<0.0001). Also, a higher proportion of participants achieved ≥5% weight loss (51.8% of individuals in the drug group versus 24% with placebo; p<0.0001). According to Feng et al. (2015) [25], liraglutide significantly reduced HbA1C level (from 8.66±2.17% at baseline to 6.92±0.95% at the end of the treatment (p<0.05).

Dulaglutide

An RCT by Frias et al. (2019) [26] showed that HbA1C reduction at the end of the treatment was significantly greater with dulaglutide 4.5 mg (-1.40±0.10% (-15.3±1.1 mmol/mol)) versus placebo (-0.44±0.10% (-4.8±1.1 mmol/mol)) (p<0.001) in addition to greater change in BW loss (-4.1±0.41 kg) versus placebo (-1.6±0.39 kg) (p<0.001). Gastrointestinal events were the most common side effect.

Taspoglutide

An RCT by Hollander et al. (2021) [31] noted a significant decrease in the mean HbA1C from baseline with taspoglutide compared to placebo (least square (LS) mean: 0.81% versus 0.09%; p<0.0001). In addition, the reduction in weight loss at week 24 was significantly greater than placebo (least square (LS) mean: 3.16 versus 1.85 kg; p<0.01). Nausea and vomiting were the most reported side effects.

Efinopegdutide

Di Prospero et al. (2021) [23] conducted an RCT that revealed that efinopegdutide significantly reduced BW at different doses (difference of LS means (95% CI) were -4.6 (-6.1, -3.1), -5.9 (-7.3, -4.4), and -7.2 (-8.7, -5.8) (p<0.001) with efinopegdutide 5 mg, 7.4 mg, and 10 mg, respectively). However, all doses showed no significant change in HbA1C and a slight numerical elevation of fasting insulin.

SGLT-1 inhibitors

Canagliflozin

Cefalu et al. (2015) [10] studied canagliflozin 100 and 300 mg for 26 weeks. The results showed significant dose-dependent reductions in BW and HbA1C with canagliflozin compared to placebo. The mean decrease in HbA1C was -0.8% and -1% with canagliflozin 100 mg and 300 mg, respectively, compared to 0.1 with placebo. The mean BW loss was -3%, 3.5%, and 0.5% with canagliflozin 100 mg, 300 mg, and placebo, respectively (p<0.001 for each).

Ertugliflozin

A study by Heymsfield et al. (2020) [29] noted that ertugliflozin 5 mg and 15 mg improved HbA1C and reduced BW significantly compared to placebo. The LS mean change (95% CI) from baseline in HbA1C was 0.1% (0%, 0.1%) for placebo, -0.8% (-0.8%, -0.7%) for ertugliflozin 5 mg, and -0.9% (-1%, -0.8%) for ertugliflozin 15 mg. In addition, the LS mean change (95% CI) in BW was -1.2 kg (-1.5, -0.9) for placebo, -3.1 kg (-3.4, -2.8) for ertugliflozin 5 mg, and -3.2 kg (-3.5, -2.9) for ertugliflozin 15 mg, respectively.

Dapagliflozin

Results from Kato et al. (2017) [32] showed that dapagliflozin caused a meaningful reduction in the levels of HbA1C, BW, blood pressure, total fat mass, and visceral fat area (VFA). However, these effects were largely reversed by the cessation of the drug. Also, changes from baseline to the end of treatment were noted in HbA1C from 8.0±1.5% to 7.3±1.3% and in BW from 81.7±17 kg to 78.5±17.8 kg (p<0.01) [32].

Empagliflozin

An RCT carried out by Neeland et al. (2016) [35] on 3,300 patients revealed that the drug significantly reduced BW compared to the placebo. The adjusted mean (95% CI) change from baseline in empagliflozin versus placebo was -1.7 kg (-2.1, -1.4 kg) and -1.9 kg (-2.1, -1.7 kg) for BW and -1.3 cm (-1.8, -0.7 cm) and -1.3 cm (-1.7, -1.0 cm) for WC, respectively (p<0.001 for each).

Licogliflozin

An RCT by He et al. (2019) [40] showed that licogliflozin 150 mg QD for 12 weeks significantly lowered BW by -6.4 kg (80% CI: -7.11, -5.72) versus 0.24 kg with placebo (80% CI: -0.46, 0.94) (p<0.0001) and improved plasma glucose level evidenced by 48% reduction in AUC0-4h following an oral glucose tolerance test (OGTT) as compared to placebo (p<0.001).

Other drug classes

Pegbelfermin (PEGylated FGF21)

The drug was evaluated by Charles et al. (2019) [21], but the results revealed no significant difference between the drug and placebo in HbA1C level and BW loss. However, significant improvements were observed in whole-body insulin sensitivity, HDL, and TG (p<0.05).

Montelukast (Leukotriene Receptor Antagonist)

El-Khateeb et al. (2023) [24] investigated the effects of montelukast with metformin. The study showed that the drug significantly improved all the measured parameters, including HbA1C and BW loss. The percent change in BW and BMI from baseline was -7.82% and -8.28%, respectively (p<0.0001). The percent change in FBG and HbA1C from baseline was -37% and -16.44%, respectively (p<0.0001).

Phentermine/Topiramate (Anorectics and Anticonvulsants)

An RCT by Garvey et al. (2014) [27] revealed that PHEN/TPM extended release (ER) and lifestyle modification could reduce both BW and blood glucose levels. A change in HbA1C level was 21.6% (217.5 mmol/mol) for PHEN/TPM ER 15/92 versus 21.2% (213.1 mmol/mol) for placebo (p=0.0381), and the change in BW was 29.4% for PHEN/TPM ER 15/92 and 22.7% for placebo (p<0.0001).

Bimagrumab (ActRII)

Heymsfield et al. (2021) [30] conducted an RCT showing that bimagrumab is effective in improving metabolic parameters, including HbA1C and BW. A change (80% CI) in HbA1C was -0.76% (-1.05%, -0.48%) in the drug group versus 0.04% (-0.23%, 0.31%) in placebo (p<0.005), and the change in BW was -5.90 kg (-7.08, -4.71) in drug study versus -0.79 kg (-1.92, 0.33) in placebo (p<0.001).

Alpha-Lipoic Acid (Caprylic Acid-Derived Antioxidant)

Okanović et al. (2014) [36] revealed that alpha-lipoic acid significantly reduced BMI and improved HbA1C and TG levels as an additive therapy. The difference in BMI from baseline to the end of treatment was -1.47 in the study group versus -0.8 in the placebo. In addition, the reduction in glucose concentration (mmol/L) was -2.36 mmol/L (p<0.001 for each).

Lorcaserin (Serotonin Receptor Agonists)

An RCT by Pi-Sunyer et al. (2016) [37] showed that lorcaserin plus diet and exercise helped promote weight loss and improve glucose control. The change in FPG was -38.1 mg/dL versus -26 mg/dL in placebo, HbA1C was -1.3% versus -1% in placebo, and BW loss was -4.5% versus -1.5% in placebo, respectively (p<0.001 for each).

Limitations

Despite these promising results, there were still some limitations. We identified a few eligible studies with small sample sizes and inadequate power, leading to imprecise estimates. Nevertheless, this systematic review was conducted comprehensively, with an appropriate number of databases searched by more than one reviewer to screen and extract data. It is based on high-quality RCTs, as indicated by the ROB assessment, which made our evidence more credible due to the quality of the data involved.

Moreover, the importance of our study lies in the clinical application of these results in the treatment of overweight/obese patients with T2DM. The addition of at least one of these drugs to the usual management of dietary and lifestyle modifications in patients with obesity and T2DM may help prevent disease progression and the various complications that accompany the disease. This opens the door for these novel treatment options to find a definitive solution for these types of patients, giving hope to millions of individuals.

Given the above, the literature surrounding the pharmacotherapy of obesity in T2DM is sparse. Consequently, one avenue of inquiry is testing the long-term side effects of these drugs. Therefore, more RCTs should investigate the therapeutic value of these medications with (1) larger sample sizes to ensure preciseness, (2) a wide range of ethnic populations, (3) study objectives assessing superiority or efficacy over placebo or standard of care (not only equivalence trials), (4) evaluation of the advantages of these drugs in each BMI subgroup, and (5) usage of intention-to-treat analysis to create results to tackle bias due to deviations from intended interventions. Furthermore, future research should consider determining the best drug and the most suitable dose with the least side effects.

## Conclusions

In conclusion, there is an urgent need for more efficacious intervention as the prevalence of T2DM and obesity are increasing in all age groups. Hence, the clinical complexity is diverse in etiology and individual response to therapies in both clinical conditions; therefore, the optimum direction is precision medicine approaches to attain the best outcome using genetic, clinical, and biochemical aspects. In addition, a combination of pharmacotherapy and lifestyle modifications is a potential intervention that could help in reducing the mortality and morbidity of diabetic obese patients.
